# Factors Associated with Urinary 1-Hydroxypyrene and Malondialdehyde among Adults near a Petrochemical Factory: Implications for Sex and Lifestyle Modification

**DOI:** 10.3390/ijerph19031362

**Published:** 2022-01-26

**Authors:** Cheng-Hsien Huang, Tung-Jung Huang, Yu-Chih Lin, Chia-Ni Lin, Mei-Yen Chen

**Affiliations:** 1 Department of Family Medicine, Chang Gung Memorial Hospital, Chiayi 613, Taiwan; shien36@cgmh.org.tw; 2 Department of Pulmonary Disease and Critical Care, Chang Gung Memorial Hospital, Yunlin 638, Taiwan; donaldhuang@cgmh.org.tw; 3 Department of Respiratory Care, Chang Gung University of Science and Technology, Chiayi 613, Taiwan; 4 Department of Family Medicine, Chang Gung Memorial Hospital, Yunlin 638, Taiwan; riverpig@cgmh.org.tw; 5 Department of Laboratory Medicine, Chang-Gung Memorial Hospital, Linkou, Taoyuan 333, Taiwan; chianilin@cgmh.org.tw; 6 Department of Medical Biotechnology and Laboratory Science, Chang Gung University, Taoyuan 333, Taiwan; 7 Department of Nursing, Chang Gung University of Science and Technology, Chiayi 613, Taiwan; 8 School of Nursing, Chang Gung University, Taoyuan 333, Taiwan; 9 Research Fellow, Department of Cardiology, Chang Gung Memorial Hospital, Chiayi 613, Taiwan

**Keywords:** 1-hydroxypyrene (1-OHP), malondialdehyde (MDA), HBsAg (hepatitis B surface antigen), anti-HCV (hepatitis C virus), petrochemical industrial complex, health promotion

## Abstract

Background: The association between the biomarkers of environmental exposure, oxidative stress, and health-related behaviors in community residents living in an endemic viral hepatitis area and near petrochemical industrial complexes remains unclear. From a health promotion perspective, healthcare providers must know what to do for residents concerned about their health and living environment, especially for individual-level and modifiable risk factors. Therefore, we aimed to explore the factors associated with urinary 1-hydroxypyrene (1-OHP) and malondialdehyde (MDA). Methods: A community-based, cross-sectional study was conducted between July 2018 and February 2019 in western coastal Yunlin County, Taiwan. All participants lived within a 10 km radius of a large petrochemical complex and did not work in the factory. This study was conducted with the local hospital through annual community health screening. Biological samples were collected and biomarkers determined and quantified in the central laboratory of the collaborating hospital. Results: A total of 6335 adult residents completed the study. The mean age was 47.7 (SD = 16) years. Out of the total population, 56.4% were female, 30.1% had metabolic syndrome (MetS), and 16.8% and 14.3% had hepatitis B virus antigen (HBsAg) and hepatitis C virus antibody (anti-HCV) positivity, respectively. The median 1-OHP and MDA level was 0.11 and 0.9 μg/g creatinine with an interquartile range of 0.07–0.18, and 0.4–1.5, respectively. The MDA levels correlated with specific diseases. The multivariable ordinal logistic regression model revealed that female sex, smoking, betel nut use, HBsAg, and anti-HCV positivity were associated with higher 1-OHP levels. In men, MetS was associated with higher 1-OHP levels and regular exercise with lower 1-OHP levels. High MDA levels were associated with smoking, betel nut users, HBsAg, and anti-HCV positivity. Conclusions: The findings highlight the importance of initiating individualized health promotion programs for residents near petrochemical factories, especially for adults with substance-use and cardiometabolic risk factors. Furthermore, it is crucial to provide further treatment to patients with viral hepatitis.

## 1. Introduction

Previous studies have indicated that petrochemical industrial complexes (PICs) have a negative effect on human health and environment, especially during the manufacturing process [1,2,3]. In recent years, many entrepreneurs of PICs have positively advanced their company’s social responsibility by decreasing and controlling emissions during the production process. In addition, the government’s environmental regulation law approaches international criteria with a higher level of air pollution restriction [4]. Public health scholars continue to monitor air quality within a 10–40 km radius from the PICs in Taiwan [3,5,6]. Hence, from a health promotion perspective, frontline healthcare providers should focus on residents living around the PICs and monitor their health and living environment. Standing with people and making efforts to empower them with a focus on individual and family holistic wellbeing are our mission.

Many studies have indicated that PICs are a source of hazardous air pollutants through the production of polycyclic aromatic hydrocarbons (PAHs). They are associated with carcinogenic properties, respiratory problems, and kidney diseases among workers in petroleum refineries, coke ovens, and aluminum production [3,7,8]. The release of hazardous substances from PICs is also thought to increase the risk of cancer and chronic diseases among people living in the neighborhood [1,2,9,10]. Many studies have shown that PAHs are produced by the incomplete combustion of organic materials, and can be metabolized as 1-hydroxypyrene (1-OHP) and excreted through the feces and urine [2,3,11]. Urinary 1-OHP has been used to assess the amount of PAH exposure. 1-OHP levels are also significantly influenced by factors that include active or passive tobacco smoking, indoor air pollution, alcohol consumption, and cooking oil fumes produced by grilled or barbecued foods [5,12,13,14,15]. Little is known about the factors associated with urine 1-OHP levels among adult residents near PICs.

A previous study revealed that the positive correlation between urinary 1-OHP and lipid peroxidation biomarkers concentration. Hence, exposure to PAHs may lead to potential oxidative stress. [16,17]. Reactive oxygen species (ROS), such as oxygen, nitrogen, and sulfur, are highly reactive derivatives of oxygen metabolism and considered part of normal cellular metabolism. ROS plays a significant role in several signaling processes and can induce cellular damage or protection [18]. Oxidation metabolites are also known as oxidative stress biomarkers. Urine malondialdehyde (MDA) is produced after the reaction of ROS with polyunsaturated fatty acids, which contain abundant double bonds and are more susceptible to ROS [19,20,21]. Urine MDA is a frequently investigated oxidative stress marker for lipid peroxidation assessment [16,22]. Many studies have indicated that MDA is correlated with aging, cancer, liver diseases, chronic obstructive pulmonary disease, and cardiometabolic diseases [23,24,25]. Aging has also been proposed to play a prominent role in oxidative stress. The relationship between oxidative stress and disease pathogenesis has been widely discussed. For instance, excess ROS are mostly derived from mitochondrial dysfunction induced by metabolic disorder-related hyperglycemia; on the other hand, hyperglycemia causes mitochondrial overproduction of ROS via greater oxygen use [22]. ROS activate the pro-inflammatory signaling pathway, and the cytokine cascades induce endothelial cell dysfunction, foamy cell formation, vascular smooth muscle migration, and hyperplasia [16,26]. Serial reactions result in atheroma formation and further cardiometabolic diseases, such as hypertension, heart disease, stroke, and diabetes [16,27]. Increased oxidative stress plays a role in metabolic syndrome, aging, and the initiation and progression of atherosclerosis [13,19]. However, adopting a balanced diet is associated with the prevention of cardiometabolic diseases, through the reduction of inflammatory biomarkers [28].

The liver is the organ with the most active catabolism and contains abundant enzymes involved in redox reactions [20,29]. The liver is also the major site for PAH catabolism. The PAHs are converted to epoxide intermediates via cytochrome P450 catalysis and then turn into ultimate carcinogens [29]. Previous studies have indicated that viral hepatitis induces extensive oxidative stress. Oxidative stress plays a pivotal role in the progression of chronic hepatitis, through lipid peroxidation, DNA oxidation, protein oxidation, ROS production, and the subsequent triggering of carcinogenesis [23,29,30]. We hypothesized that urine 1-OHP and MDA are associated with an unhealthy lifestyle and chronic viral hepatitis due to inflammation. Previous studies have shown that the coastal city of Yunlin, Taiwan has a high prevalence of residents who engage in alcohol consumption, betel nut chewing, cigarette smoking, inadequate dietary adoption, and low exercise, and live around an endemic viral hepatitis region [31,32,33]. This phenomenon was because, five decades ago, many rural residents of Taiwan were treated with inadequately disinfected medical equipment while sick. For instance, they were given injections by unqualified physicians, which caused many innocent people to be unknowingly infected by HCV [32,33]. Therefore, the aim of this study was to explore the factors associated with urinary 1-OHP and MDA levels among adult residents near large PICs in central southwestern Taiwan.

## 2. Materials and Methods

### 2.1. Study Design and Population

We conducted a community-based, cross-sectional study between July 2018 and February 2019 in Yunlin County. All participants lived within a 10 km radius of a large petrochemical factory for many years. Considering the independent age and ability to participate in this study, the inclusion criteria were (1) area of residence in Mailiao and Taihsi designated townships; (2) more than 20 years of age and not working inside the PICs; (3) able to communicate in Mandarin or Taiwanese; (4) able to walk or drive to the local hospital and community health activity center; and (5) agreeing to participate in this study and signing the informed consent form. The exclusion criteria were an inability to answer questions or incomplete data.

### 2.2. Procedure and Ethical Approval

This study was approved by the institutional review board of the research ethics committee (No: IRB 201800428B0). It was conducted in collaboration with a local hospital through annual community health screening. All procedures were performed in accordance with the Helsinki Declaration. The township heads sent messages (oral and poster) regarding free health check-ups and invited individuals to participate in this study. Six nursing students were recruited as research assistants and trained by the investigators. The research team described the study procedures (e.g., collecting blood and urine specimens and an interview to track health-related habits with a questionnaire). The questionnaire consisted of health-related lifestyle behaviors, which were designed by the research team and based on previous studies [31,32]. Blood samples were drawn 8 h after fast; blood and urine samples were stored and sent to the central laboratory of the cooperating hospital.

### 2.3. Measurements

This study collected the following information via individual interviews and questionnaires along with the physiological biomarkers:

Demographic characteristics and health-related lifestyle behaviors, including sex, age, educational level (years), body height (cm), and body weight (kg). Participants were asked to report the frequency of seven health habits: (e.g., how often do you have…?): regular alcohol consumption (never vs. current or former user), betel nut chewing (never vs. current or former user), cigarette smoking (never vs. current or former smoker), at least three servings of vegetables (1.5 bowls) per day, two servings of fruit (1 bowl) per day, at least 1500 mL of water per day, and at least 30 min of exercise (3 times per week). The research team determined the frequencies of eating proper servings of vegetables/fruit/water and of adopting exercise as low (never/seldom) or often (usually/always).

Metabolic syndrome (MetS) and serum biomarkers: Based on the national standard [34], participants with the presence of three abnormal cardiometabolic risk factors, out of five, were classified as having MetS: (1) central obesity: waist circumference > 90/80 cm in males and females, measured between the last rib margin and the iliac crest; (2) systolic/diastolic blood pressure ≥ 130/85 mmHg; (3) high-density lipoprotein cholesterol < 40/50 mg/dL in males/females; (4) fasting blood glucose ≥ 100 mg/dL; (5) triglyceride level ≥ 150 mg/dL. Liver health information, including alanine aminotransferase (ALT > 35 U/L) and aspartate aminotransferase (AST > 35 U/L) levels, was used to detect liver inflammation status. Hepatitis B virus (surface antigen) and serum anti-hepatitis C virus (HCV) antibody (positive or negative) were used to identify viral hepatitis. Hepatitis B surface antigen (HBsAg) was analyzed by enzyme-linked immunosorbent assay (ELISA) using a SURASE B-96 plate (General Biological Corp., Hsinchu, Taiwan). Anti-HCV antibody was assessed by ELISA using an SP-NANBASE C-96 3.0 plate (General Biological Corp. Hsinchu, Taiwan).

Urine 1-OHP and MDA: Spot urine samples were collected and stored below −80 °C until urine 1-OHP and MDA levels were quantified in the central laboratory of the collaborating hospital. Both 1-OHP and MDA were expressed as μg/g CRE. Urinary 1-OHP was analyzed using ultra-performance liquid chromatography-tandem mass spectrometry (UPLC-MS/MS). Urinary MDA was quantified using the standard thiobarbituric acid reactive substances (TBARS) assay. Urinary creatinine concentration was used for urinary 1-OHP and MDA adjustments.

### 2.4. Statistical Analyses

Because there were no reference values for urine 1-OHP and MDA concentrations, we equally divided the participants into quartiles according to 1-OHP and MDA levels. The demographics and characteristics among the MDA or 1-OHP quartiles were compared using one-way analysis of variance for continuous variables and a chi-square test for categorical variables, with a Bonferroni multiple comparison when the overall test was significant. The linear trend of the demographics and characteristics across the MDA or 1-OHP quartiles was also tested for linear contrast in a general linear model for continuous variables and the Cochran–Armitage test for categorical variables. Factors associated with higher levels of 1-OHP or MDA were investigated using a multivariable odds proportional model (ordinal logistic regression). The analysis was performed on the entire cohort and stratified by sex. MetS and body mass index (BMI) components were not included in the multivariable model due to collinearity with MetS. Laboratory data for liver function (AST and ALT) were also not entered into the multivariable model due to the collinearity with HBsAg and anti-HCV. Finally, the relationship between 1-OHP and MDA levels was examined using the Spearman’s rank correlation. All tests were two-tailed, and *p* < 0.05 was considered statistically significant. Data analyses were conducted using SPSS 25 (IBM SPSS Inc., Chicago, IL, USA).

## 3. Results

### 3.1. Participant Demographic Characteristics

A total of 6335 participants aged ≥ 20 years who completed the examination were included in the study, of whom 3574 (56.4%) were female. The mean age was 47.7 years, and the average education level was 9.6 years. Approximately half of the subjects (55.5%) were overweight or obese and MetS was prevalent in 30.1% of the population (*n* = 1909). Of note, 16.8% (*n* = 1067) and 14.3% (*n* = 909) of the subjects were identified as HBsAg and anti-HCV positive, respectively. The median 1-OHP level was 0.11 μg/g creatinine, with an interquartile range (IQR) of 0.07 to 0.18. The median MDA level was 0.9 μg/g creatinine, with an IQR of 0.4 to 1.5 (Table 1).

**Figure 1:**
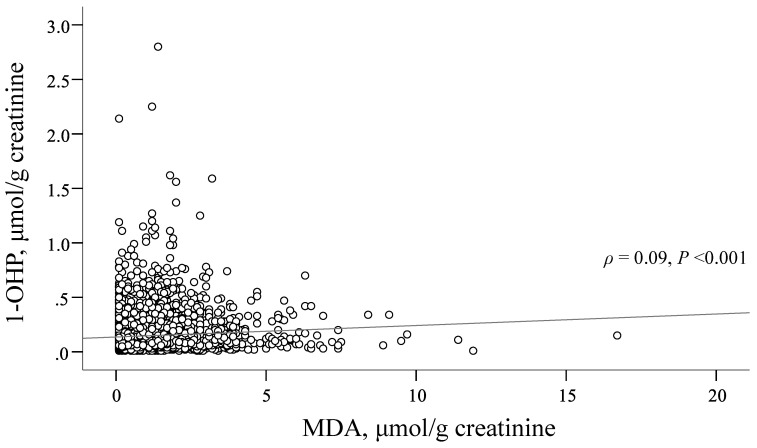
Relationship between MDA and 1-OHP levels. MDA, malondialdehyde; 1-OHP, 1-hydroxypyrene.

Compared to male subjects, females tended to have an older age, less education, lower prevalence of MetS, lower likelihood of substance use (smoking, alcohol drinking, and betel nut chewing), better dietary habits, and less exercise. Females also tended to have a lower prevalence of HBsAg and better liver function, but a higher potential for HCV infection. The results also showed that female subjects had slightly higher 1-OHP levels and slightly lower MDA levels than male subjects (Table 1). Additionally, the relationship between 1-OHP and MDA levels was examined (Figure 1). The results showed that the correlation was significantly positive, but weak (correlation coefficient = 0.09).

### 3.2. Factors Associated with 1-OHP Level

According to the quartiles of 1-OHP, Table 2 shows that there were significant differences in age, sex, education, BMI, MetS and its components (except waist circumference), substance use, dietary habits, regular exercise, HBsAg, anti-HCV, and liver function. Based on the multivariable ordinal logistic regression model, Table 3 demonstrates that older age (odds ratio (OR) 0.97, 95% confidence interval (CI) 0.96–0.98) and a higher education level (OR 0.96, 95% CI 0.95–0.97) were associated with a lower 1-OHP level. In contrast, several factors were identified to be associated with a higher 1-OHP level, including female (OR 2.30, 95% CI 2.06–2.57), smoking (OR 6.94, 95% CI 5.96–8.08), betel nut chewing (OR 1.25, 95% CI 1.04–1.52), HBsAg (OR 1.27, 95% CI 1.12–1.43), and anti-HCV positivity (OR 1.43, 95% CI 1.12–1.43). After stratifying the analysis by sex, the results revealed that MetS was associated with a higher 1-OHP level in male subjects (OR 1.17, 95% CI 1.00–1.36) but not in female subjects. In addition, an association between frequent regular exercise and lower 1-OHP levels was observed only in male participants.

### 3.3. Factors Associated with MDA Level

Based on the MDA level quartiles, Table 4 shows that there were significant differences in age, sex, education, BMI, MetS and its components, substance use, dietary habit of vegetable (but not fruit) consumption, both types of hepatitis, and liver function. Based on the multivariable ordinal logistic regression model, Table 5 demonstrated that older age (OR 1.02, 95% CI 1.01–1.02), smoking (OR 1.32, 95% CI 1.15–1.51), betel nut chewing (OR 1.22, 95% CI 1.02–1.45), HBsAg (OR 1.25, 95% CI 1.11–1.41), and anti-HCV positivity (OR 1.48, 95% CI 1.29–1.70) were associated with a higher MDA level. In contrast, a higher education level was associated with a lower MDA level (OR 0.98, 95% CI 0.97–0.99). After stratifying the analysis by sex, the results were generally consistent between male and female participants. The effect of smoking and betel nut chewing in male subjects was more apparent because the prevalence of betel nut chewing was higher in men.

## 4. Discussion

Our results provide important data for further study and evidence-based lifestyle modifications. This study had three important findings. First, smoking, betel nut chewing, HBsAg, and anti-HCV positivity were significantly associated with higher urinary concentrations of 1-OHP and MDA. Second, a high prevalence of cardiometabolic risk factors and chronic viral hepatitis was found in this population. Third, sex differences were found in adopting unhealthy lifestyles, and men tended to show substance use and inadequate diet, while females tended to take less regular exercise.

The present findings strongly indicate an association between smoking and urinary 1-OHP concentration, with an odds ratio of 6.94, which was statistically significant in women and men, and is in agreement with the findings of previous studies [12,13,35,36]. This is not surprising, because each cigarette contains 3.2–16.0 ng benzo(a)pyrene [37]. A substantial amount of evidence supports the suggestion that active or passive cigarette smoking-related oxidative stress causes inflammation, which in turn results in further generation of ROS and potentially increases oxidative damage to macromolecular targets that may lead to cancer initiation and/or progression [35,38]. This also explains why smokers tended to have higher urinary MDA levels, with an odds ratio of 1.32. The betel nut (also called areca) has been confirmed as a class I carcinogen and is a popular addictive substance in Asia [31]. Furthermore, some studies have confirmed that areca nut extract and other areca ingredients can induce ROS in oral epithelial cells, which may involve oxidative stress via redox imbalance [39]. Our study revealed that betel nut users had higher 1-OHP and MDA concentrations, with 1.25 and 1.22 odds ratios, respectively. This implies that the influence of betel nut-induced oxidative stress may not be confined to the oral cavity. Notably, previous studies found that cigarette smokers also tended to have betel nut-chewing and alcohol-drinking habits in this study area [31]. The present finding also indicates that education level was significantly associated with lower 1-OHP and MDA levels. A higher education level may enable individuals to adopt healthier lifestyles, including having a balanced diet, less substance use, and more regular exercise. Therefore, further studies are needed to initiate smoking and betel nut cessation programs, especially for community adults with lower socioeconomic status.

Theoretically, adopting adequate exercise and consuming fruits or vegetables rich in dietary antioxidants vitamin C, vitamin E, and carotenoids may lower oxidative stress and result in lower 1-OHP and MDA biomarker levels, as supported by the findings of many studies [12,28]. However, this benefit was not consistent with the findings of the present study. This could be because nutrients were quantified using a simple questionnaire, which might have caused a distortion of the real data or the dietary amount. Studies have shown that it is difficult to demonstrate the benefits of nutrient intervention in nutrient-deficient populations [20]. Therefore, it may not be feasible to manage low oxidative stress therapy using nutrient supplements. The present findings show that adopting regular exercise in male adults resulted in significant benefits in lowering 1-OHP concentration, with an odds ratio of 0.8, but did not influence the MDA biomarker. Hence, quantitation of specific types of exercise and diets should be clarified in further studies, to determine the most effective practices for relieving oxidative stress.

According to a report from the Nutrition and Health Survey in Taiwan (NAHSIT) 2005–2008, the prevalence of adult MetS was 25.5% and increased from 13.6% in NAHSIT 1993–1996 [34]. The present study demonstrated that 30.1% of the participants had MetS and 55.5% were overweight or obese, and these frequencies were significantly higher than those observed nationwide and in Asian regions [40]. Since ROS can initiate the inflammatory signaling pathway, in which cytokines induce endothelial cell dysfunction, the serial reactions would result in atheroma formation and other cardiometabolic diseases [22,27]. Hence, fundamental prevention strategies should focus on reducing MetS and cardiometabolic risks through lifestyle modification, while considering sex differences [41]. For instance, we should empower male adults to adopt a healthier diet, with sufficient servings of vegetables and fruit, and female adults to perform regular exercise.

The current prevalence of HCV infection in Taiwan is approximately 4% [42]. A high prevalence of viral hepatitis was noted in the present study. This may be related to an incident which happened a few decades ago, where blood-borne virus transmission was caused by inadequate sterilization of medical equipment, or infection by unscreened blood products in Taiwan, specifically in the southwestern coastal area [42,43]. Previous studies have indicated that oxidative stress, viral hepatitis, and liver cancer may be interconnected [23,29]. HCV has evolved to manipulate pro- and antioxidant balance and generate sustainable oxidative stress that not only causes hepatic damage but also stimulates processes that reduce damage treatment. Lipid metabolism pathways present a clear danger to the accumulation of viral-induced ROS. These disorders tend to lead to cirrhosis and hepatocellular carcinoma (HCC), but there is little real-world data confirmation. Our large-scale data verified this theory and demonstrated that viral hepatitis carriers are prone to higher levels of 1-OHP and MDA, especially chronic hepatitis C carriers. Oxidative stress may trigger HCC in patients with chronic viral hepatitis [20,30]. Therefore, oxidative biomarker assessment may provide us with a better understanding of disease history and progress beyond traditional laboratory studies. Fortunately, the WHO has launched a global health strategy to eliminate hepatitis C by 2030 [30,43], and the Taiwanese government has implemented an elimination goal, with free antiviral treatment by National Health Insurance [42,44]. Recently, the emergence of direct-acting antivirals, such as asunaprevir/daclatasvir, has increased cure rates and treatment safety, with more than 95% efficiency, few adverse effects, and a short treatment time of 8–12 weeks [44,45]. Many adults near the PICs with chronic viral hepatitis do not receive the much-needed available information [32]. Therefore, it is crucial for us to recommend both HBV and HCV carriers for further antiviral treatment.

Compared with females, male sex was significantly associated with a higher prevalence of smoking, alcohol consumption, and betel nut chewing, and tended to also be associated with more regular exercise but improper diet habits, in agreement with published findings [31,33]. The present findings showed that the prevalence of current or former female smokers was only 4.5%, and this rate was lower than that of males (40.5%). However, the female sex was associated with significantly higher levels of urinary 1-OHP. This result is consistent with those of previous studies [8,11,12]. This could be due to the amount of time women spend in the kitchen, and cooking methods with frying and high temperatures can produce cooking oil fumes. In addition, poorly ventilated kitchens may increase fume inhalation [13,41]. The 1-OHP is a sensitive biomarker that reflects indoor air pollutant exposure [15]. Exposure to cooking oil fumes can lead to disturbed cardiovascular autonomic function and an increased risk of oxidative DNA injury [5]. Furthermore, numerous studies have reported an increase in the incidence of cardiovascular and respiratory diseases among women and children who lived around smokers [46,47]. As a cohort study in the United States, Gearhart-Serna et al. [36] indicated that smoking and secondhand smoke exposure accounted for the largest PAH intake, and that there were strong interactions between race/ethnicity and smoking or second-hand smoke, especially in vulnerable populations for high PAH exposure. From the perspectives of human and environmental health, it is necessary to conduct a tailored health promotion program, including strategies to reduce cooking oil fumes by improving ventilation devices and methods to avoid secondhand smoke. In addition, it is time to initiate more aggressive actions in the smoking cessation program and regulation for the vulnerable group near the PICs.

There are some limitations to this study. First, the limited geographical scope might limit the generalizability of the findings. Second, the health-related behavior assessments were mostly self-reported and were not measured more precisely, for example, water or vegetable intake, although we demonstrated servings with a standardized container. Third, due to the lack of information on the history of medications used for hypertension or diabetes, an underestimation of the prevalence of cardiometabolic risks might have occurred.

## 5. Conclusions

This study used large-scale data to explore the association between the biomarkers of environmental exposure, oxidative stress, and health-related behaviors in community residents living in an endemic area of viral hepatitis and nearby large PICs. A high prevalence of cardiometabolic risk factors and chronic viral hepatitis was found in this population. Furthermore, smoking, betel nut use, HBsAg, and anti-HCV positivity were associated with higher urinary 1-OHP and MDA levels. The findings highlight the importance of initiating individualized health promotion programs for adult residents, including cardiometabolic risk prevention, and smoking and betel-nut cessation, and regulations to avoid second-hand smoke. In addition, there is an urgent need to provide antiviral treatment to residents with chronic hepatitis.

## Figures and Tables

**Table 1:** Demographic characteristics of the study subjects according to gender (*N* = 6335).

Variable	Total (*N* = 6335)	Female (*n* = 3574)	Male (*n* = 2761)	*p*-Value
Age, year	47.7 ± 16.1	47.9 ± 16.2	47.3 ± 16.0	0.109
Education level, years	9.6 ± 5.8	8.8 ± 6.2	10.7 ± 4.9	<0.001
Body mass index ≥ 24 kg/m^2^	3518 (55.5)	1672 (46.8)	1846 (66.9)	<0.001
Metabolic syndrome (MetS)	1909 (30.1)	980 (27.4)	929 (33.6)	<0.001
No. of MetS components	1.74 ± 1.39	1.59 ± 1.42	1.93 ± 1.33	<0.001
Each component of MetS				
SBP/DBP ≥ 130/85 mmHg	3468 (54.7)	1703 (47.6)	1765 (63.9)	<0.001
Waist circumference ^1^	2636 (41.6)	1518 (42.5)	1118 (40.5)	0.113
FBG ≥ 100 mg/dL	2217 (35.0)	1096 (30.7)	1121 (40.6)	<0.001
HDL-C < 40/50 (M/F) mg/dL	778 (12.3)	210 (5.9)	568 (20.6)	<0.001
Triglyceride ≥ 150 mg/dL	1213 (19.1)	460 (12.9)	753 (27.3)	<0.001
Smoking				<0.001
Never	5055 (79.8)	3412 (95.5)	1643 (59.5)	
Current/Quit	1280 (20.2)	162 (4.5)	1118 (40.5)	
Alcoholic drinking				<0.001
Never	5520 (87.1)	3472 (97.1)	2048 (74.2)	
Current/Quit	815 (12.9)	102 (2.9)	713 (25.8)	
Betel nut chewing				<0.001
Never	5699 (90.0)	3544 (99.2)	2155 (78.1)	
Current/Quit	636 (10.0)	30 (0.8)	606 (21.9)	
Intake vegetables				<0.001
Never/Seldom	2144 (33.8)	1056 (29.5)	1088 (39.4)	
Often	4191 (66.2)	2518 (70.5)	1673 (60.6)	
Intake fruit				<0.001
Never/Seldom	3512 (55.4)	1823 (51.0)	1689 (61.2)	
Often	2823 (44.6)	1751 (49.0)	1072 (38.8)	
Adopt regular exercise				<0.001
Never/Seldom	4392 (69.3)	2593 (72.6)	1799 (65.2)	
Often	1943 (30.7)	981 (27.4)	962 (34.8)	
HBsAg	1067 (16.8)	528 (14.8)	539 (19.5)	<0.001
Anti-HCV	909 (14.3)	553 (15.5)	356 (12.9)	0.004
AST > 35 U/L	653 (10.3)	277 (7.8)	376 (13.6)	<0.001
ALT > 35 U/L	1362 (21.5)	461 (12.9)	901 (32.6)	<0.001
1-OHP, μg/g CRE	0.11 [0.07, 0.18]	0.11 [0.07, 0.17]	0.10 [0.06, 0.19]	0.015
MDA, μg/g CRE	0.90 [0.40, 1.50]	0.90 [0.40, 1.50]	1.00 [0.50, 1.50]	0.034

Abbreviations: MetS, metabolic syndrome; SBP, systolic blood pressure; DBP, diastolic blood pressure; FBG, fasting blood glucose; HDL-C, high-density lipoprotein cholesterol; M, male; F, female; HBsAg, hepatitis B surface antigen; HCV, hepatitis C virus; AST, aspartate aminotransferase; ALT, alanine aminotransferase; 1-OHP, 1-hydroxypyrene; CRE, creatinine; MDA, malondialdehyde; ^1^ Waist circumference, male > 90 cm and female > 80 cm; data are presented as frequency (percentage), mean ± standard deviation or median [25th, 75th percentile].

**Table 2:** Factors associated with the quartiles of urine 1-OHP concentration level (*N* = 6335).

Variable	Q1 (*n* = 1864)	Q2 (*n* = 1483)	Q3 (*n* = 1462)	Q4 (*n* = 1526)	*p*-Value	*p* Trend
Level, μmol/g CRE ^1^	≤0.07	0.07–0.11	0.11–0.18	>0.18		
Age, year	50.7 ± 17.2	47.3 ± 16.4 ^a^	46.1 ± 15.8 ^a^	45.8 ± 14.2 ^a^	<0.001	<0.001
Female	946 (50.8)	874 (58.9) ^a^	956 (65.4) ^a,b^	798 (52.3) ^b,c^	<0.001	0.014
Education level, years	9.4 ± 6.3	9.9 ± 6.0	9.7 ± 5.7	9.4 ± 5.0	0.064	0.867
BMI ≥ 24 kg/m^2^	1079 (57.9)	828 (55.8)	767 (52.5) ^a^	844 (55.3)	0.020	0.035
MetS	581 (31.2)	433 (29.2)	412 (28.2)	483 (31.7)	0.117	0.970
Each component of MetS	1.83 ± 1.39	1.66 ± 1.38 ^a^	1.66 ± 1.38 ^a^	1.78 ± 1.41	0.001	0.335
Each MetS component						
SBP/DBP ≥ 130/85 mmHg	1162 (62.3)	788 (53.1) ^a^	753 (51.5) ^a^	765 (50.1) ^a^	<0.001	<0.001
WC ^1^	749 (40.2)	633 (42.7)	613 (41.9)	641 (42.0)	0.489	0.335
FBG ≥ 100 mg/dL	732 (39.3)	499 (33.6) ^a^	456 (31.2) ^a^	530 (34.7) ^a^	<0.001	0.001
HDL-C < 40/50(M/F) mg/dL	216 (11.6)	147 (9.9)	157 (10.7)	258 (16.9) ^a,b,c^	<0.001	<0.001
TG ≥ 150 mg/dL	352 (18.9)	234 (15.8)	261 (17.9)	366 (24.0) ^a,b,c^	<0.001	<0.001
Smoking					<0.001	<0.001
Never	1696 (91.0)	1322 (89.1)	1197 (81.9) ^a,b^	840 (55.0) ^a,b,c^		
Current/Quit	168 (9.0)	161 (10.9)	265 (18.1) ^a,b^	686 (45.0) ^a,b,c^		
Alcoholic drinking					<0.001	<0.001
Never	1703 (91.4)	1321 (89.1)	1284 (87.8) ^a^	1212 (79.4) ^a,b,c^		
Current/Quit	161 (8.6)	162 (10.9)	178 (12.2) ^a^	314 (20.6) ^a,b,c^		
Betel nut chewing					<0.001	<0.001
Never	1743 (93.5)	1392 (93.9)	1334 (91.2) ^b^	1230 (80.6) ^a,b,c^		
Current/Quit	121 (6.5)	91 (6.1)	128 (8.8) ^b^	296 (19.4) ^a,b,c^		
Intake vegetables					<0.001	<0.001
Never/Seldom	589 (31.6)	464 (31.3)	508 (34.7)	583 (38.2) ^a,b^		
Often	1275 (68.4)	1019 (68.7)	954 (65.3)	943 (61.8) ^a,b^		
Intake fruit					<0.001	<0.001
Never/Seldom	985 (52.8)	784 (52.9)	811 (55.5)	932 (61.1) ^a,b,c^		
Often	879 (47.2)	699 (47.1)	651 (44.5)	594 (38.9) ^a,b,c^		
Adopt regular exercise					<0.001	<0.001
Never/Seldom	1211 (65.0)	1004 (67.7)	1055 (72.2) ^a,b^	1122 (73.5) ^a,b^		
Often	653 (35.0)	479 (32.3)	407 (27.8) ^a,b^	404 (26.5) ^a,b^		
HBsAg	275 (14.8)	238 (16.0)	270 (18.5) ^a^	284 (18.6) ^a^	0.005	0.001
Anti-HCV	247 (13.3)	214 (14.4)	204 (14.0)	244 (16.0)	0.147	0.043
AST > 35 U/L	159 (8.5)	133 (9.0)	148 (10.1)	213 (14.0) ^a,b,c^	<0.001	<0.001
ALT > 35 U/L	379 (20.3)	291 (19.6)	310 (21.2)	382 (25.0) ^a,b^	0.001	0.001

Abbreviations: CRE, Creatinine; BMI, body mass index; 1-OHP, 1-hydroxypyrene; Q, quartile; SBP, systolic blood pressure; DBP, diastolic blood pressure; FBG, fasting blood glucose; HDL-C, high-density lipoprotein cholesterol; TG, triglyceride; HBsAg, hepatitis B surface antigen; HCV, hepatitis C virus; AST, aspartate aminotransferase ; ALT, alanine aminotransferase; ^1^ Waist circumference, male > 90 cm and female > 80 cm; ^a^, ^b^, and ^c^ indicate significant difference as compared to the Q1, Q2, and Q3 groups, respectively, by using Bonferroni post hoc test.

**Table 3:** Association between the demographic characteristics and the risk of higher level of 1-OHP.

Explanatory Variable	Total		Female		Male	
	Adjusted OR (95% CI)	*p*-Value	Adjusted OR (95% CI)	*p*-Value	Adjusted OR (95% CI)	*p*-Value
Age, year	0.97 (0.96–0.98)	<0.001	0.97 (0.96–0.98)	<0.001	0.98 (0.97–0.98)	<0.001
Female	2.30 (2.06–2.57)	<0.001	-	-	-	-
Education level, years	0.96 (0.95–0.97)	<0.001	0.96 (0.95–0.98)	<0.001	0.94 (0.93–0.96)	<0.001
Metabolic syndrome	1.10 (0.99–1.22)	0.082	1.05 (0.90–1.21)	0.561	1.17 (1.00–1.36)	0.049
Frequent intake vegetables	1.00 (0.90–1.11)	0.990	1.06 (0.92–1.22)	0.457	0.97 (0.83–1.13)	0.665
Frequent intake fruit	0.96 (0.87–1.07)	0.465	1.02 (0.89–1.16)	0.766	0.86 (0.73–1.01)	0.060
Adopt regular exercise	0.93 (0.84–1.03)	0.179	1.09 (0.95–1.25)	0.237	0.80 (0.69–0.94)	0.005
Smoking	6.94 (5.96–8.08)	<0.001	9.27 (6.46–13.30)	<0.001	6.01 (5.06–7.15)	<0.001
Alcoholic drinking	1.16 (0.99–1.36)	0.061	1.20 (0.82–1.75)	0.347	1.13 (0.95–1.35)	0.162
Betel nut chewing	1.25 (1.04–1.52)	0.019	2.04 (1.01–4.10)	0.046	1.14 (0.93–1.39)	0.204
HBsAg	1.27 (1.12–1.43)	<0.001	1.26 (1.07–1.49)	0.007	1.30 (1.09–1.55)	0.003
Anti-HCV	1.43 (1.24–1.64)	<0.001	1.49 (1.24–1.79)	<0.001	1.39 (1.11–1.73)	0.003

Abbreviations: 1-OHP, 1-hydroxypyrene; HBsAg, hepatitis B surface antigen; HCV, hepatitis C virus; OR, odds ratio; CI, confidence interval.

**Table 4:** Factors associated with the quartiles of urine MDA level (*N* = 6335).

Variable	Q1 (*n* = 1654)	Q2 (*n* = 1581)	Q3 (*n* = 1601)	Q4 (*n* = 1499)	*p*-Value	*p* Trend
Level, μmol/g creatinine	≤0.4	0.4–0.9	0.9–1.5	>1.5		
Age, year	43.7 ± 15.3	44.7 ± 15.5	48.3 ± 15.5 ^a,b^	54.4 ± 16.1 ^a,b,c^	<0.001	<0.001
Female	1022 (61.8)	839 (53.1) ^a^	826 (51.6) ^a^	887 (59.2) ^b,c^	<0.001	0.058
Education level, years	10.8 ± 5.5	10.7 ± 5.5	9.5 ± 5.6 ^a,b^	7.3 ± 6.0 ^a,b,c^	<0.001	<0.001
Body mass index ≥ 24 kg/m^2^	814 (49.2)	833 (52.7)	941 (58.8) ^a,b^	930 (62.0) ^a,b^	<0.001	<0.001
Metabolic syndrome (MetS)	398 (24.1)	433 (27.4)	502 (31.4) ^a^	576 (38.4) ^a,b,c^	<0.001	<0.001
No. of MetS components	1.51 ± 1.32	1.63 ± 1.40	1.81 ± 1.40 ^a,b^	2.04 ± 1.40 ^a,b,c^	<0.001	<0.001
Each component of MetS						
SBP/DBP ≥ 130/85 mmHg	818 (49.5)	814 (51.5)	907 (56.7) ^a,b^	929 (62.0) ^a,b,c^	<0.001	<0.001
Waist circumference ^1^	607 (36.7)	612 (38.7)	675 (42.2) ^a^	742 (49.5) ^a,b,c^	<0.001	<0.001
FBG ≥ 100 mg/dL	487 (29.4)	499 (31.6)	583 (36.4) ^a,b^	648 (43.2) ^a,b,c^	<0.001	<0.001
HDL-C < 40/50 (M/F) mg/dL	153 (9.3)	187 (11.8)	210 (13.1) ^a^	228 (15.2) ^a,b^	<0.001	<0.001
Triglyceride ≥ 150 mg/dL	258 (15.6)	295 (18.7)	348 (21.7) ^a^	312 (20.8) ^a^	<0.001	<0.001
Smoking					<0.001	<0.001
Never	1400 (84.6)	1266 (80.1) ^a^	1219 (76.1) ^a,b^	1170 (78.1) ^a^		
Current/Quit	254 (15.4)	315 (19.9) ^a^	382 (23.9) ^a,b^	329 (21.9) ^a^		
Alcoholic drinking					<0.001	0.002
Never	1481 (89.5)	1385 (87.6)	1354 (84.6) ^a^	1300 (86.7)		
Current/Quit	173 (10.5)	196 (12.4)	247 (15.4) ^a^	199 (13.3)		
Betel nut chewing					<0.001	<0.001
Never	1543 (93.3)	1449 (91.7)	1404 (87.7) ^a,b^	1303 (86.9) ^a,b^		
Current/Quit	111 (6.7)	132 (8.3)	197 (12.3) ^a,b^	196 (13.1) ^a,b^		
Intake vegetables					0.078	0.017
Never/Seldom	591 (35.7)	552 (34.9)	515 (32.2)	486 (32.4)		
Often	1063 (64.3)	1029 (65.1)	1086 (67.8)	1013 (67.6)		
Intake fruit					0.382	0.125
Never/Seldom	934 (56.5)	894 (56.5)	869 (54.3)	815 (54.4)		
Often	720 (43.5)	687 (43.5)	732 (45.7)	684 (45.6)		
Adopt regular exercise					0.071	0.018
Never/Seldom	1168 (70.6)	1110 (70.2)	1115 (69.6)	999 (66.6)		
Often	486 (29.4)	471 (29.8)	486 (30.4)	500 (33.4)		
HBsAg	235 (14.2)	247 (15.6)	299 (18.7) ^a^	286 (19.1) ^a^	<0.001	<0.001
Anti-HCV	164 (9.9)	153 (9.7)	225 (14.1) ^a,b^	367 (24.5) ^a,b,c^	<0.001	<0.001
AST > 35 U/L	105 (6.3)	134 (8.5)	175 (10.9) ^a^	239 (15.9) ^a,b,c^	<0.001	<0.001
ALT > 35 U/L	293 (17.7)	317 (20.1)	377 (23.5) ^a^	375 (25.0) ^a,b^	<0.001	<0.001

Abbreviations: MDA, malondialdehyde; Q, quartile; SBP, systolic blood pressure; DBP, diastolic blood pressure; FBG, fasting blood glucose; HDL-C, high-density lipoprotein cholesterol; HBsAg, hepatitis B surface antigen; HCV, hepatitis C virus; AST, aspartate aminotransferase ; ALT, alanine aminotransferase; ^1^ Waist circumference, male > 90 cm and female > 80 cm; ^a^, ^b^, and ^c^ indicate significant difference as compared to the Q1, Q2, and Q3 groups, respectively, by using Bonferroni post hoc test.

**Table 5:** Association between the demographics/characteristics and the risk of higher level of MDA.

Explanatory Variable	Total		Female		Male	
	Adjusted OR (95% CI)	*p*-Value	Adjusted OR (95% CI)	*p*-Value	Adjusted OR (95% CI)	*p*-Value
Age, year	1.02 (1.01–1.02)	<0.001	1.02 (1.01–1.03)	<0.001	1.02 (1.01–1.03)	<0.001
Female	0.99 (0.89–1.10)	0.792	-	-	-	-
Education level, years	0.98 (0.97–0.99)	0.001	0.99 (0.97–1.01)	0.217	0.96 (0.94–0.98)	<0.001
Metabolic syndrome	1.08 (0.98–1.20)	0.129	1.13 (0.97–1.31)	0.113	1.06 (0.91–1.22)	0.482
Frequent intake vegetables	1.08 (0.97–1.19)	0.173	1.14 (0.99–1.31)	0.074	1.01 (0.87–1.18)	0.884
Frequent intake fruit	1.00 (0.91–1.10)	0.994	0.96 (0.84–1.09)	0.518	1.05 (0.90–1.22)	0.517
Adopt regular exercise	1.01 (0.91–1.11)	0.914	0.98 (0.85–1.12)	0.756	1.05 (0.90–1.21)	0.551
Smoking	1.32 (1.15–1.51)	<0.001	1.30 (0.97–1.74)	0.079	1.33 (1.14–1.56)	<0.001
Alcoholic drinking	1.00 (0.86–1.16)	0.956	1.18 (0.82–1.70)	0.381	0.96 (0.81–1.14)	0.637
Betel nut chewing	1.22 (1.02–1.45)	0.029	1.10 (0.55–2.21)	0.780	1.23 (1.01–1.49)	0.039
HBsAg	1.25 (1.11–1.41)	<0.001	1.25 (1.06–1.47)	0.009	1.28 (1.08–1.52)	0.004
Anti-HCV	1.48 (1.29–1.70)	<0.001	1.43 (1.19–1.72)	<0.001	1.56 (1.26–1.94)	<0.001

Abbreviations: MDA, malondialdehyde; HBsAg, hepatitis B surface antigen; HCV, hepatitis C virus; OR, odds ratio; CI, confidence interval.

## Data Availability

The individual-level data used and/or analyzed for the current study are available from the corresponding author on request.

## Abbreviations

PICs: petrochemical industrial complexes; 1-OHP, 1-hydroxypyrene; MDA, malondialdehyde; MetS, metabolic syndrome; HBsAg, hepatitis B surface antigen; HCV, hepatitis C virus; SBP, systolic blood pressure; DBP, diastolic blood pressure; FBG, fasting blood glucose; HDL-C, high-density lipoprotein cholesterol; AST, aspartate aminotransferase; ALT, alanine aminotransferase; WC, waist circumference; ROS, reactive oxygen species; TBARs, thiobarbituric acid reactive substances; IQR, inter-quartile range.
